# Study on the Photocatalytic and Antibacterial Properties of TiO_2_ Nanoparticles-Coated Cotton Fabrics

**DOI:** 10.3390/ma12122010

**Published:** 2019-06-23

**Authors:** Guangyu Zhang, Dao Wang, Jiawei Yan, Yao Xiao, Wenyan Gu, Chuanfeng Zang

**Affiliations:** 1 National & Local Joint Engineering Research Center of Technical Fiber Composites for Safety and Health, School of Textile and Clothing, Nantong University, Nantong 226019, China; wdaojs@163.com (D.W.); xyt123asd@163.com (Y.X.); mrsgu1@sina.com (W.G.); 2 College of Chemical and Biological Engineering, Zhejiang University, Hangzhou 310027, China; 3 Faculty of Textile Science and Technology, Shinshu University, 3-15-1, Tokida, Ueda, Nagano 386-8567, Japan; 19fs110c@shinshu-u.ac.jp

**Keywords:** amino-capped TiO_2_ NPs, cotton fabric, photocatalytic degradation, antibacterial properties

## Abstract

Herein, the amino-capped TiO_2_ nanoparticles were synthesized using tetrabutyl titanate and amino polymers by a two-step sol-gel and hydrothermal method technique for the fabrication of functional cotton fabric. The prepared TiO_2_ nanoparticles and the treated cotton fabric were characterized by transmission electron microscope (TEM), X-ray diffraction (XRD), field emission scanning electron microcopy (FE-SEM) photocatalytic and antibacterial measurement. The results indicate the typical characteristic anatase form of the amino-capped TiO_2_ NPs with an average crystallite size of 14.9 nm. The treated cotton fabrics exhibit excellent antibacterial property and good photocatalytic degradation of methylene blue.

## 1. Introduction

Cotton as an important natural renewable cellulose fiber has good hygroscopicity and breath ability, which is widely used in clothing, home textiles, medical materials and other fields. Despite the excellent properties of cotton, some inherent features such as being poor persistence to ultraviolet (UV) radiation inhibited its wide application in advanced fields. In recent years, functional finishing of cotton has attracted the greatest attention. Nanomaterials can impart new functional properties to cotton fiber for its applications, such as antimicrobial properties [1], ultraviolet (UV) protection [2,3], superhydrophobicity [4], self-cleaning [5,6,7] and flame retardancy [8]. 

As one of the most important nano-inorganic material, titanium dioxide nanoparticles (TiO_2_ NPs) possesses efficient photo catalyst activity and chemical stability [9]. It is widely used in antibacterial, anti-ultraviolet materials [10,11,12], self-cleaning glass [13], lithium batteries [14], photocatalysts and paints [15,16]. TiO_2_ NPs prepared by sol-gel method have good uniformity, high purity, small particle size and simple preparation process, which can simultaneously improve the UV protection performance and photocatalytic efficiency of fabric. For instance, Dao and Xin reported the nucleation and successful growth of anatase crystallites on cotton fabrics. Cotton fabric coatings with TiO_2_ exhibit good anti-UV, antibacterial and self-cleaning properties [17]. Li and Zhu prepared TiO_2_/Ag through a two-step process. The functionalized cotton fabrics were imparted with UV protection and antibacterial properties [10]. However, TiO_2_ NPs with small particle size and large specific surface area are easily adsorbed to form aggregates. TiO_2_ nanoparticles (NPs) lack chemical bonds to link with fibers, which will bring about unsatisfied durability. Therefore, some binders, such as citric acid (CA), polyurethane resin and polyacrylic esters (PALS), were required to fix the nanoparticles on fibers to provide durability of functional properties. Moreover, numerous chemicals used in preparation of nanoparticles and the finishing process may be associated with environmental toxicity or require complex processes as well as high-energy consumption and costs, making them unsuitable for large-scale production [18].

In our previous study, an amino-hyperbranched polymer (HSDA) was synthesized. It was utilized to control synthesis of Ag and ZnO nanoparticles, and also served as a binder to impart and fix the nanoparticles on cellulose fabric to provide antimicrobial properties [19,20,21]. In this paper, amino-capped TiO_2_ NPs were attempted to be synthesized by the sol-gel and hydrothermal method, using a mixture of tetrabutyl titanate (TBT)and amino polymers as a raw material. In the process of sol-gel formation of TiO_2_ NPs, hyperbranched polymer can control the particle size of TiO_2_ NPs and improve the dispersion uniformity of nanoparticles to prevent its agglomeration. The prepared TiO_2_ NPs were characterized by transmission electron microscope (TEM), X-ray diffraction (XRD) and ultraviolet-visible spectra (UV-vis) measurement. Then, amino-capped TiO_2_ NPs were coated on cotton fabric by the impregnation method. As cotton fiber contains a large amount of -OH, thus the amino-capped TiO_2_ NPs can be firmly combined with the fiber through hydrogen bonding and van der Waals force. The antibacterial properties and photocatalytic efficiency of TiO_2_ NPs-coated cotton fabric were investigated, the comparison of similar research is listed in Table S1.

## 2. Materials and Methods

### 2.1. Materials

Cotton fabric with a basic weight of 100 g/m^2^ was obtained from Hua Fang Co., Ltd. (Suzhou, China). Tetrabutyl titanate (TBT, 98%) and ethanol (99.7%) were purchased from Guoyao Chemical Technology Co., Ltd. (Shanghai, China). Amino polymers were prepared as described in our previous paper [19].

### 2.2. Synthesis of Amino-Capped TiO_2_ NPs

The amino polymer-modified nano-TiO_2_ sol was prepared by a one-step sol-gel method at 25 °C under vigorous magnetic stirring, an ethanol solution containing 5 mL of TBT was added dropwise to 12 mL of ethanol solution containing amino polymers (0.5 g), deionized water (3 mL) and glacial acetic acid (pH 3 to 4). The mixed solution was continuously stirred to form a pale yellow three-dimensional network gel. Afterward, a mixture of absolute ethanol (10 mL) and deionized water (15 mL) were added dropwise. Stirring was continued until the mixture was homogeneous. The sol was transferred to a high-temperature hydrothermal Teflon reaction vessel, sealed in a steel cup and heated in an oven at 240 °C for 10 h. The obtained TiO_2_ NPs were washed with deionized water, centrifuged and dried by grinding to obtain the amino-capped TiO_2_ NPs fine powders.

### 2.3. Preparation of TiO2 NPs-Coated Cotton Fabric

A cotton fabric weighing 5 g was immersed in a 20 g/L NaOH solution at a bath ratio of 1:50. The mixture was shaken in a water bath at 60 °C for 1 h. The cotton fabric was washed with deionized water and dried for use. The amino-capped TiO_2_ NPs were dispersed in 100 mL water with a concentration of 2 g/L. The alkali-treated cotton fabric was immersed in the amino-capped TiO_2_ NPs dispersion solution at a bath ratio of 1:30 and shaken in a water bath at 60 °C for 2 h. The TiO_2_ NPs-coated cotton fabric was thermally pre-treated at 80 °C for 5 min and then at 150 °C for 3 min [22,23].

The amino polymer with a concentration of 0.5 g/L was prepared, and the cotton fabric was treated in the amino solution as contrast.

### 2.4. Characterization of the Amino-Capped TiO_2_ NPs and Treated Cotton Fabric

The morphology and lattice characteristics of the amino-capped TiO_2_ NPs were characterized by TEM (JEOL, Tokyo, Japan). The crystalline phase of TiO_2_ NPs and the treated fabric were analyzed by XRD, (Philips, Amsterdam, The Netherlands) via a Cu Kα X-ray light source at a voltage of 40 kV and a current of 30 mA. The light absorption properties of TiO_2_ NPs were analyzed by UV-vis spectra (UV-3010 Hitachi spectrophotometer, Tokyo, Japan). The surface morphology of cotton fiber was characterized by field emission scanning electron microcopy (FE-SEM) (Scios Dual-Beam, Brno, Czech Republic) and energy dispersive spectrometer (EDS) (Carl Zeiss, EVO15, Oberkochen, Germany). The antibacterial activity of amino-treated fabric and TiO_2_ NPs-coated fabric against *Escherichia coli* (*E. coli*) and *Staphylococcus aureus* (*S. aureus*) was tested according to GB/T 20944.3-2008 (China) using the shake flask method. 0.75 g of the pristine fabric sample was cut into pieces of about 0.5 × 0.5 cm^2^ size, and immersed in a flask containing 70 mL of 0.3 mM phosphate buffer saline medium with a concentration of 1 × 10^5^ CFU/mL–4 × 10^5^ CFU/mL. The flask was then shaken on a rotary shaker at 150 rpm for 18 h at 24 °C. One mL of the solution was taken from each of the incubated samples, diluted and dispensed onto agar plates. All plates were incubated at 37 °C for 24 h and the formed colonies were counted. The photocatalytic of the amino-capped TiO_2_ NPs were evaluated by degradation of methylene blue under ultraviolet irradiation, the light flux in this experiment was 300 mW/cm^2^ and the reaction vessel was 10 cm. The amino-capped TiO_2_ NPs-coated cotton fabric of 1 g and a 200 mL methylene blue solution (5 mg/L) were placed in the GHX-3 type photochemical reaction apparatus. The as-prepared samples were taken every 30 min. Under the same conditions, the amino- treated cotton fabric was evaluated as contrast. After the supernatant was taken and centrifuged, the absorbance of the methylene blue solution at 664 nm was measured by an UV-visible spectrophotometer. The photocatalytic degradation rate of methylene blue solution in the presence of amino-capped TiO_2_ NPs-coated cotton fabric was calculated as:

η(%) = 1 − A_t_/A_0_ (1)

where η is the photocatalytic degradation rate; A_0_ and A_t_ are the absorbance of the methylene blue solution before and after degradation, respectively.

## 3. Results

### 3.1. Synthesis and Characterization of Amino-Capped TiO_2_ NPs

**Scheme 1:**
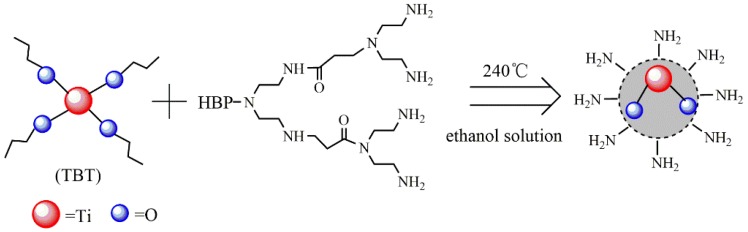
Schematic illustration of synthesis of amino-capped TiO_2_ nanoparticles (NPs).

The principle of synthesizing the amino-capped TiO_2_ NPs is described in Scheme 1. The solution of amino polymer was added into the TBT/ethanol solution by hydrothermal reaction. The TiO_2_ colloids prepared in acidic media were adsorbed strongly onto the TiO_2_ surface. The amino served as a dispersing agent and stabilizer to prevent the agglomeration of TiO_2_ NPs [24]. Upon hydrothermal dissolution-recrystallization reaction, the amino-capped TiO_2_ NPs were obtained.

**Figure 1:**
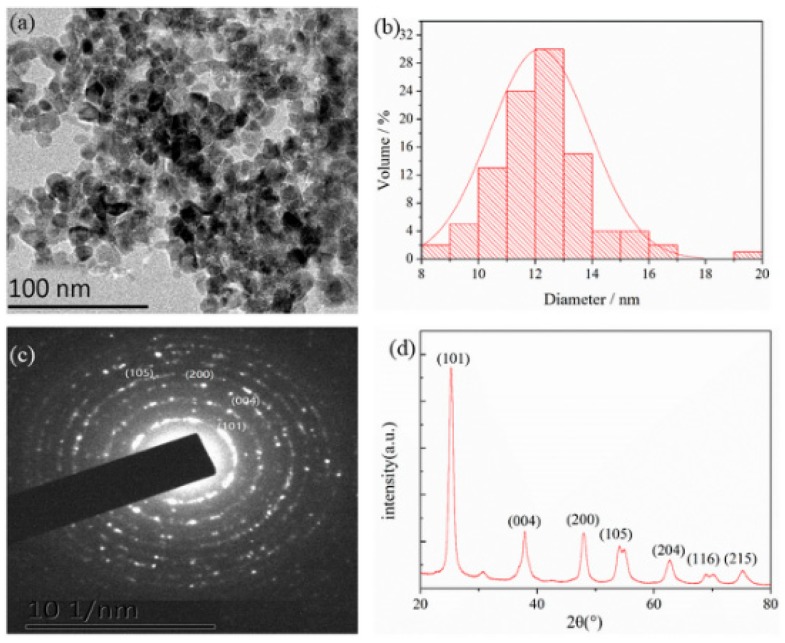
(**a**) TEM images, (**b**) size distribution, (**c**) selected area electron diffraction (SAED), (**d**) XRD of amino-capped TiO_2_ NPs.

The prepared TiO_2_ NPs were observed by the TEM and XRD measurement. Figure 1a shows the TEM image of the amino-capped TiO_2_, much more grains with tetragonal shape can be seen. The diameter of amino-capped TiO_2_ NPs was found about 12 nm in Figure 1b. Series of diffraction rings appeared in the selected area electron diffraction (SAED) pattern, as presented in Figure 1c, indicating the pure anatase crystalline phase in the amino-capped TiO_2_ NPs.

**Figure 2:**
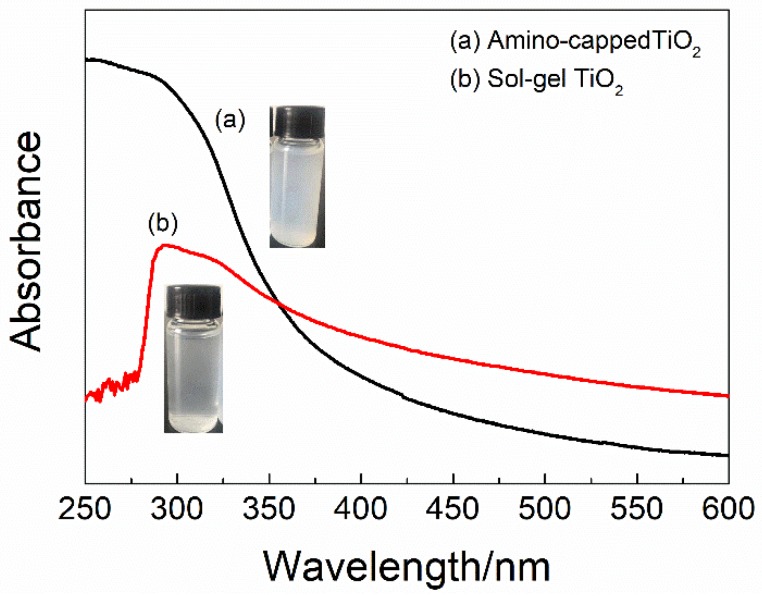
UV-vis spectra of (**a**) TiO_2_ and (**b**) amino-capped TiO_2_ colloidal solution.

The crystalline phase of the amino-capped TiO_2_ NPs was characterized by the powder XRD method shown in Figure 1d, with the XRD pattern similar to that of anatase TiO_2_ (JCPDS No. 21-1272). After the hydrothermal process, the amino-capped TiO_2_ NPs actively formed well crystalline TiO_2_ NPs and completed phase change under the control of amino. Reflection peaks are observed at 2θ values of 25.26, 37.8, 47.96, 54.14, 54.94, 62.76, 68.94, 70.12, and 75.12, indexed to diffraction peaks from the (101), (004), (200), (105), (211), (204), (116), (220), and (215) planes of anatase crystalline phase of TiO_2_ [24,25]. Obviously, the amino-capped TiO_2_ NPs exhibit an anatase phase structure and extremely small particle sizes. The UV-vis absorption properties of TiO_2_ NPs and amino-capped TiO_2_ NPs were investigated by UV-Vis spectroscopy, with results shown in Figure 2. The absorption at 320 nm corresponds to the bandgap of the anatase TiO_2_ (3.2 eV) [26], which requires UV radiation (λ < 387 nm) to transfer an electron from valence to conduction band. As TiO_2_ NPs has poor solubility, it shows very faint shades in the aqueous solution. The TiO_2_ NPs that were capped by amino can enhance the solubility, which exhibited a transparent blue color in the aqueous solution (inset of Figure 2). Compared with the pure TiO_2_ (Figure 2a), the amino-capped TiO_2_ NPs (Figure 2b) absorb more ultraviolet light in the region of 200 nm to 400 nm, which demonstrates that the amino-capped TiO_2_ NPs possess good UV absorption intensity for UV light [27].

### 3.2. Preparation and Characterization of the Cotton Fabric Coated by the Amino-Capped TiO_2_ NPs

**Figure 3:**
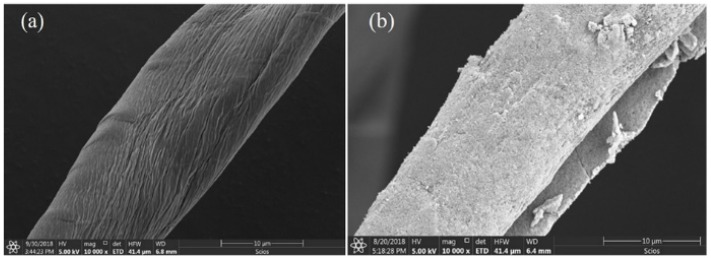
SEM images of (**a**) cotton fiber, (**b**) TiO_2_ NPs-coated cotton fiber.

As TiO_2_ has large surface energy it is difficult to disperse and combine it with cotton fibers. To provide the TiO_2_ NPs-coated cotton fabric and impart the cotton fabric with photocatalytic activity and antibacterial properties, the cotton fabric was treated with the aqueous solution of amino-capped TiO_2_ NPs by the impregnation method. The surface morphology of cotton fabric and TiO_2_ NPs-treated cotton fabric was observed by FE-SEM, as displayed in Figure 3a, the pure cotton fiber has wrinkles and a smooth surface. The TiO_2_ NPs-coated cotton fiber presenting obvious differences, which were covered with a large quantity of TiO_2_ NPs and the nanoparticles were well-dispersed.

**Scheme 2:**
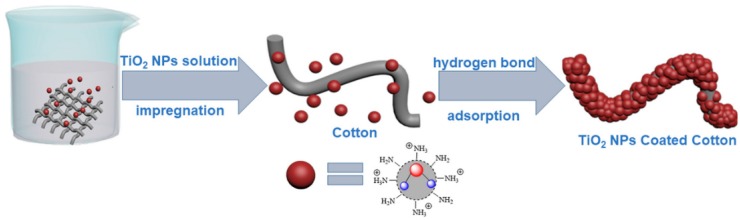
Schematic illustration of TiO_2_ NPs-coated cotton fiber.

The mechanism of the evenly dispersed TiO_2_ NPs on cotton fabric is provided in Scheme 2. TiO_2_ NPs shows the positive charge as it was capped by the amino polymer shown in Figure S1. In the amino-capped TiO_2_ NPs solution, TiO_2_ NPs can be easily combined with cotton fabric through intermolecular hydrogen bonds between amino end groups and pendent hydroxyl groups on cellulose fiber. As the side-chain OH groups of cotton fiber can be typically ionized in the aqueous phase and the cotton fiber carries certain negative surface charges. Electrostatic bonding interactions between the negatively charged hydroxyl groups on cellulose fiber and positively charged amino end groups can enhance the stability of amino-capped TiO_2_ NPs on the surface of cotton fabric [28].

**Figure 4:**
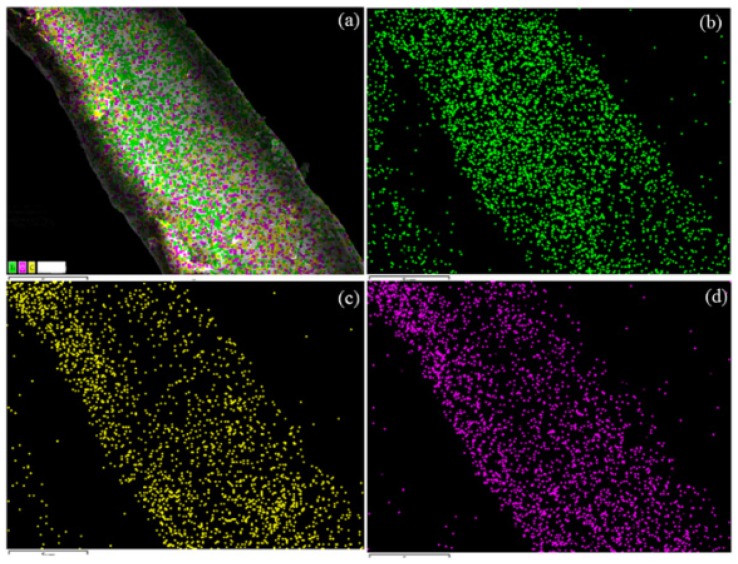
EDS (energy dispersive spectrometer) images of amino-capped TiO_2_ NPs grafted cotton fabric: (**a**) cotton, (**b**) Ti, (**c**) O, (**d**) C.

**Figure 5:**
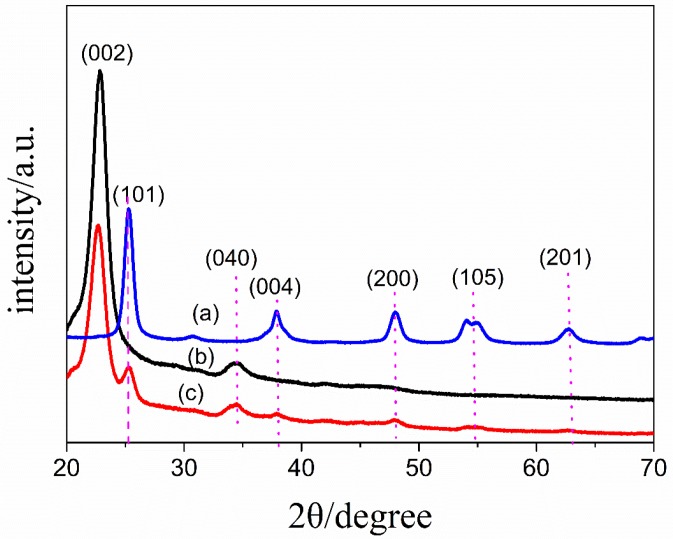
XRD patterns of (**a**) anatase TiO_2_, (**b**) cotton fabric and (**c**) TiO_2_ NPs-coated cotton fabric.

The elementals of the treated cotton fabric were further investigated using the EDS mapping method. As shown in Figure 4a and Figure S1, additional Ti elements on the surface of the cotton fabric are observed. From Figure 4b–d, O and C elements of the cellulose fiber, as well as the even distribution of Ti on the fabric surface are found. The result is in good agreement with FE-SEM measurement. To determine the TiO_2_ NPs-coated on cotton fabric, the XRD patterns of the control and treated cotton fabrics were tested, as shown in Figure 5. The diffraction peaks at 2θ values of 22.82 and 34.26 are shown in Figure 5a,b, which are assigned to the (002) and (040) planes of cellulose fiber. In contrast, the treated cotton fiber has reflection peaks at 2θ values of 25.28, 37.92, 47.92, which are indexed to the (101), (004), (200) planes of the anatase phase shown in Figure 5c. The results confirm that the amino-capped TiO_2_ NPs were coated on the cotton fiber effectively [10,24].

### 3.3. Photocatalytic Activity of the TiO_2_ NPs-Coated Cotton Fabric

**Figure 6:**
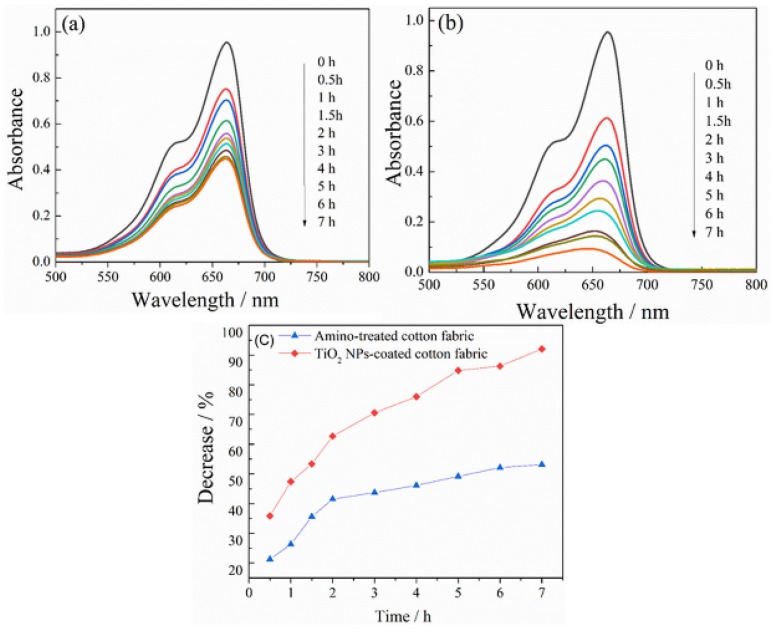
The absorbance of methylene blue by (**a**) amino-treated cotton fabric, (**b**) TiO_2_ NPs-coated cotton fabric under UV-light, and (**c**) the decrease rate of methylene blue by amino-treated and TiO_2_ NPs-coated cotton fabric.

Photocatalytic experiments were implemented to investigate the photocatalytic activity of the amino-treated and TiO_2_ NPs-coated cotton fabrics. The cotton fabrics were firstly dipped in methylene blue solution in dark condition to examine the adsorption property. Amino-treated and TiO_2_ NPs-coated cotton fabrics have the similar adsorption capacity shown in Figure S3, they can absorb about 25% of the methylene blue in solution. Figure 6a shows the absorbance of methylene blue solution added with amino-treated cotton fabric with time under UV light irradiation. A medium change of absorbance value and a slight impact of the UV light on the degradation rate of methylene blue are observed. Figure 6b shows the gradual decrease in the absorbance of methylene blue solution added with amino-capped TiO_2_ NPs-coated cotton fabric. It indicates that the TiO_2_ NPs-coated cotton fabric degraded the methylene blue solution. Figure 6c shows the decrease rate of methylene blue solution under UV light irradiation. After 7 h, the decrease rate of methylene blue solution reached 53.14%, and that of TiO_2_ NPs-coated cotton fabric was up to 92.03%.

**Scheme 3:**
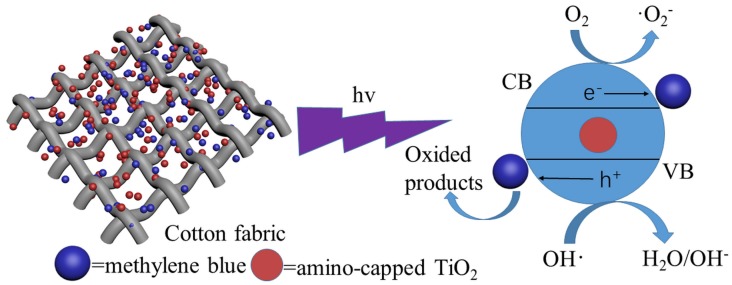
Proposed mechanism of photocatalytic degradation of methylene blue by amino-capped TiO_2_ NPs under UV illumination.

The mechanism of photocatalytic activity and self-cleaning properties (Figure S4) of the TiO_2_ NPs-coated cotton fabric was illustrated in Scheme 3. The amino-capped TiO_2_ NPs on cotton fabric could adsorb the methylene blue molecules in the solution. When the light energy is equal to or greater than the band gap energy, a high concentration of conduction-band electrons (e^−^) and valence-band holes (h^+^) is generated in the density of TiO_2_ nanoparticles on cotton fabric. Photogenic positive hole (h^+^) reacts with hydroxyl group (–OH) and/or adsorbed water molecules produces hydroxyl radicals (•OH), which act as strong oxidants during the photocatalytic reaction. Furthermore, photo generated electrons (e^−^) react with an electron acceptor, such as O_2_ and are adsorbed on the surface of the catalyst or dissolved in water to produce superoxide radical anions O_2_• and •HO_2_. Free radicals react with each other, leading to formation of hydrogen peroxide and increasing gaseous oxygen in the photocatalytic reaction. [25,29,30,31] All this react with methylene blue molecules for their full degradation.

**Figure 7:**
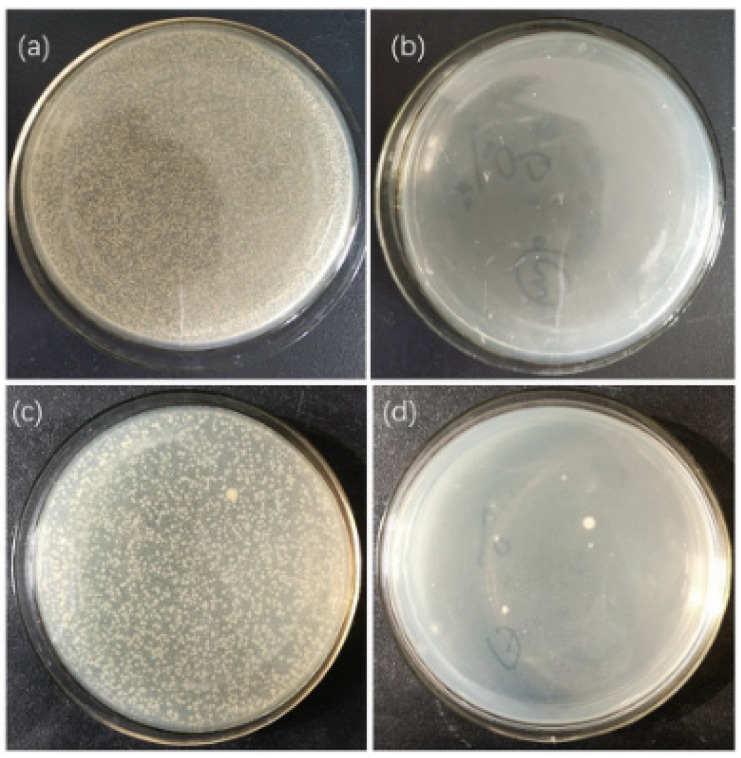
*S. aureus* colonies of (**a**) amino-treated (**b**) TiO_2_ NP_S_-coated cotton fabric and *E. coli* colonies of (**c**) amino-treated (**d**) TiO_2_ NP_S_-coated cotton fabric.

The antibacterial activity of cotton fabric was qualitatively evaluated by the shake-flask method under visible light. The comparison results between *E. coli* and *S. aureus* bacteria cells between amino-treated and TiO_2_ NPs-coated cotton fabric were presented in Figure 7. As presented in Figure 7a.b, bacteria had grown all over the plate, meaning that amino-treated fabric did not show antibacterial activities against *S. aureus* and *E. coli* colonies. By contrast, TiO_2_ NPs-coated cotton fabric exhibited excellent antibacterial activities because nearly no bacteria grew in the plate, as shown in Figure 7b,d. The bacterial reduction rate of both *S. aureus* and *E. coli* can reach more than 99%. Both *S. aureus* and *E. coli* exhibits negative charge, the positively charged amino can adsorb the bacteria on the surface of TiO_2_ by electrostatic adherence. As reported, TiO_2_ has good photocatalytic property under ultraviolet conditions, it can generate OH radicals. Due to the electron mediator transports between cells and TiO_2_, OH radicals destroyed bacterial cells caused by reduction of coenzyme cell content [32]. Moreover, TiO_2_ NPs are known to inactivate bacteria by binding to electron-donating groups and cause little pores in bacterial cell walls, leading to increased permeability and cell death [33]. In this research, amino-capped TiO_2_ NPs have good photocatalytic activity and adsorption property attributed to their high UV absorption intensity (Figure 2), this can endow that amino-capped TiO_2_ has good antibacterial properties under visible light. 

## 4. Conclusions

In this study, the amino-modified anatase TiO_2_ nanoparticles with a morphology size of 12 nm were synthesized by a simple sol-gel method using amino polymer. Due to the numerous amino groups, TiO_2_ NPs were adsorbed on cotton fabric. The results of FE-SEM and XRD confirm that the amino-capped TiO_2_ NPs were well dispersed. Amino-capped TiO_2_ NPs-coated cotton fabric shows great photo degradation of MB under UV irradiation, with the photocatalytic degradation and adsorption rate of 92.03%. In addition, as-prepared cotton fabric exhibits good antibacterial properties under visible light, the bacterial reduction rate of both *S. aureus* and *E. coli* can reach more than 99%.

## Supplementary Materials

The following are available online at https://www.mdpi.com/1996-1944/12/12/2010/s1, Figure S1: EDS analyze of amino-capped TiO_2_ NPs on cotton fabric; Figure S2: FTIR spectra of (a) amino-capped TiO_2_, (b) amino polymers; Figure S3: Absorbance of methylene blue (a) absorbance of methylene blue by impregnation of amino treated fabric (b) and TiO_2_ NPs coated fabric (c) without UV-light in 7 h; Figure S4: Self-cleaning properties of (a) cotton fabric (b) TiO_2_ NPs-coated cotton fabric with UV light; Table S1: Comparison of published data with our research.
