# Supplementary material for: Study on the Photocatalytic and Antibacterial Properties of TiO2 Nanoparticles-Coated Cotton Fabrics
Source: Materials (Basel). 2019 Jun 23;12(12):2010. doi: 10.3390/ma12122010 (PMC6630916; doi:10.3390/ma12122010)
Supplement: Supplementary file 1 [file materials-12-02010-s001.pdf]

Supplementary Materials: Study on the Photocatalytic and Antibacterial Properties of TiO_2 Nanoparticles-Coated Cotton Fabrics

Guangyu Zhang [1,2,*], Dao Wang [1], Jiawei Yan [3], Yao Xiao [1], Wenyan Gu [1] and Chuanfeng Zang [1]

Table S1. Comparison of published data with our research.

Materials	Photocatalytic Efficiency	Antibacterial Properties		Reference
		S. aureus	*E. coli*	
TiO_2/cotton	/	86%	86%	[1]
TiO_2/SiO_2/cotton	/		36%	[2]
TiO_2/cotton	/		85.8%	[3]
TiO_2/cotton	/	73.5%	75.15%	[4]
TiO_2/cotton	/	26.14%	9.55%	[5]
TiO_2/chitosan/cotton	/	7.9 mm	7.75 mm	[6]
TiO_2/MB	95%			[7]
TiO_2/MB	90%			[8]
TiO_2/MB	pH 7: 85%			[9]
TiO_2/cotton/tea stain	K/S: 0	99.9%	99.9%	[10]
TiO_2/cotton/amino	>92%	99.9%	99.9%	our paper

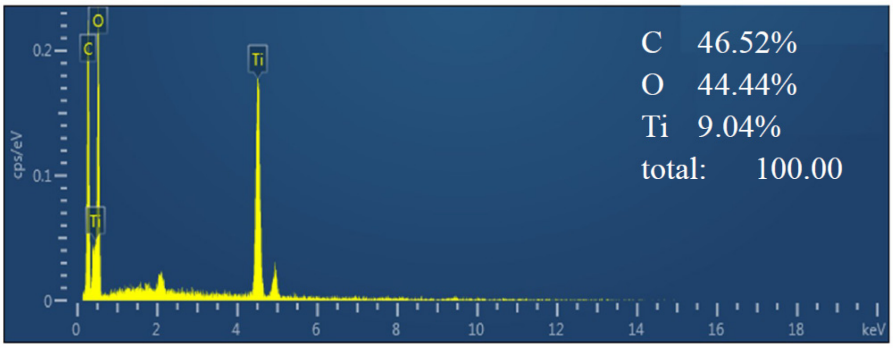

Figure S1. EDS analyze of amino-capped TiO_2 NPs on cotton fabric.

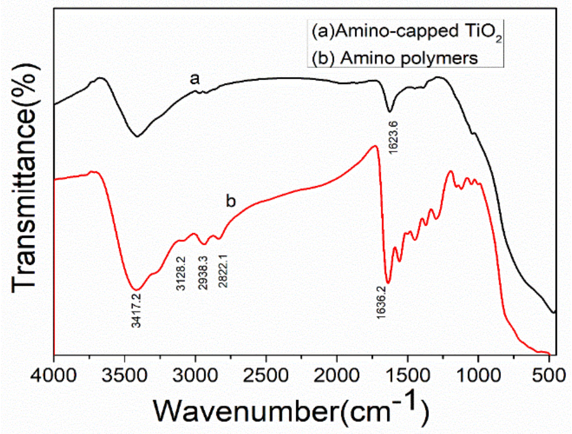

Figure S2. FTIR spectra of (**a**) amino-capped TiO_2, (**b**) amino polymers.

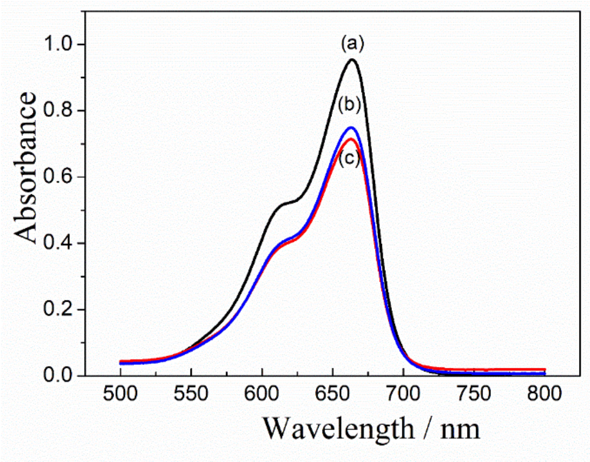

Figure S3. Absorbance of methylene blue (**a**) absorbance of methylene blue by impregnation of amino treated fabric (**b**) and TiO_2 NPs coated fabric (**c**) without UV-light in 7 h.

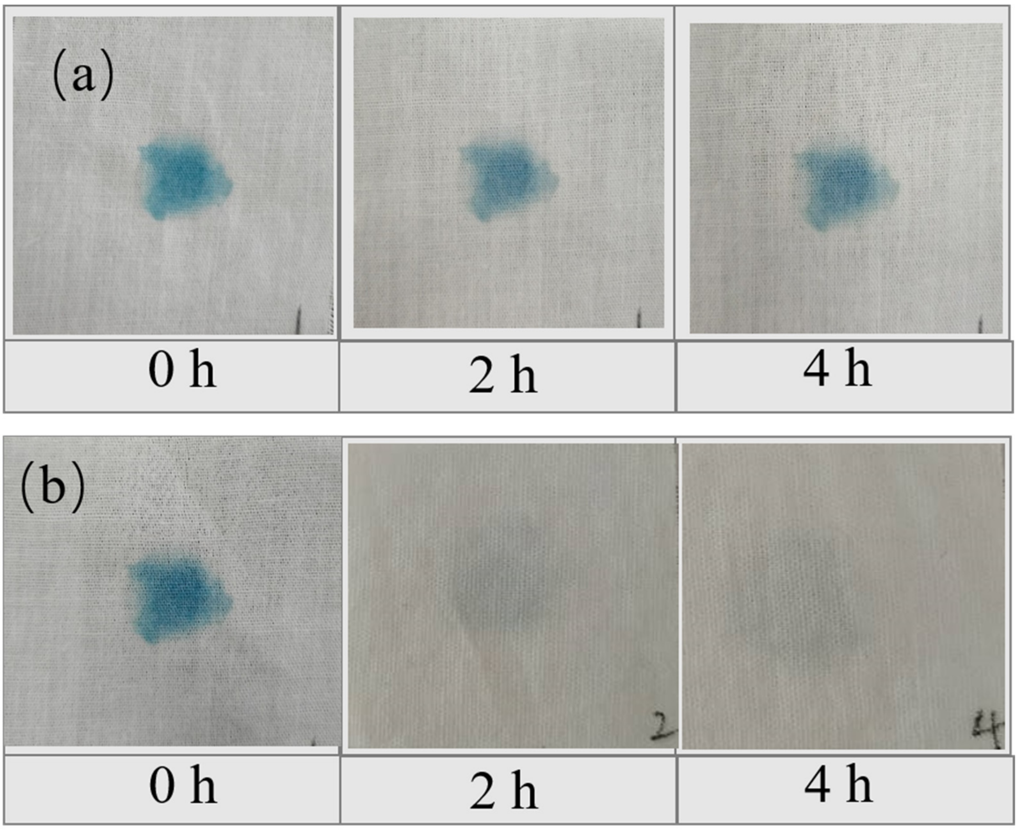

Figure S4. Self-cleaning properties of (**a**) cotton fabric (**b**) TiO_2 NPs-coated cotton fabric with UV light.

Reference

1. Li, S.; Zhu, T.; Huang, J.; Guo, Q.; Chen, G.; Lai, Y. Durable antibacterial and UV-protective Ag/TiO_2@ fabrics for sustainable biomedical application. *Int. J. Nanomed.* **2017**, *12*, 2593.
2. Rajendran, V.; Dhineshbabu, N.R.; Kanna, R.R.; Kaler, K.V. Enhancement of thermal stability, flame retardancy, and antimicrobial properties of cotton fabrics functionalized by inorganic nanocomposites. *Ind. Eng. Chem. Res.* **2014**, *5*, 19512–19524.
3. Deng, H.; Ren, W.; Huang, Z.L.; Xiao, C.F. Synthesis of Nanoscale Iodine Doped TiO_2 and Properties Treated Cotton Fabric. *J. Mater. Eng. 2008*, *10*, 60–63.

4. Huang, Y.; Wang, L.M.; Shen, Y. Application of Ag-Doped Nano-TiO_2 Prepared at Low-Temperature to Multi-Functional Finishing of Cotton Fabric. *Adv. Mater. Res.* **2013**, *821*, 1346–1352.
5. Li, J.; Li, R.; Du, J.; Ren, X.; Worley, S.D.; Huang, T.S. Improved UV stability of antibacterial coatings with N-halamine/TiO_2. *Cellulose* **2013**, *20*, 2151–2161.
6. Rilda, Y.; Safitri, R.; Agustien, A.; Nazir, N.; Syafiuddin, A.; Nur, H. Enhancement of antibacterial capability of cotton textiles coated with TiO_2–SiO_2/chitosan using hydrophobization. *J. Chin. Chem. Soc.* **2017**, *64*, 1347–1353.
7. Guillard, C.; Puzenat, E.; Lachheb, H. Why inorganic salts decrease the TiO_2 photocatalytic efficiency. *J. Alloys Compd.* **2007**, *503*, 485–489.
8. Wang, L.; Shen, Y.; Xu, L. Thermal crystallization of low-temperature prepared anatase nano-TiO_2 and multifunctional finishing of cotton fabrics. *J. Text. Instit. Proc. Abstracts* **2016**, *107*, 12.
9. Liu, Q.Y.; Li, J.; Cao, J.; Li, C.R. Synthesis of Composite TiO_2/Natural Cotton Cellulose Nanofiber with Photocatalytic Property. *Appl. Mech. Mater.* **2014**, *665*, 393–396
10. Doganli, G.; Yuzer, B.; Aydin, I.; Gultekin, T.; Con, A.H.; Selcuk, H.; Palamutcu, S. Functionalization of cotton fabric with nanosized TiO_2 coating for self-cleaning and antibacterial property enhancement. *Journal Coat. Technol. Res.* **2016**, *13*, 257–265.
